# Mycorrhizal Fungi as Bioprotectors of Crops Against Verticillium Wilt—A Hypothetical Scenario Under Changing Environmental Conditions

**DOI:** 10.3390/plants9111468

**Published:** 2020-10-30

**Authors:** Nieves Goicoechea

**Affiliations:** Plant Stress Physiology Group, Department of Environmental Biology, School of Sciences, Universidad de Navarra, Associated to CSIC (EEAD, Zaragoza, ICVV, Logroño), 31008 Pamplona, Spain; niegoi@unav.es; Tel.: +34-9-4842-5600 (ext. 806489)

**Keywords:** arbuscular mycorrhizal fungi, biocontrol, climate change, soil-borne pathogens, *Verticillium* spp.

## Abstract

The association that many crops can establish with the arbuscular mycorrhizal fungi (AMF) present in soils can enhance the resistance of the host plants against several pathogens, including *Verticillium* spp. The increased resistance of mycorrhizal plants is mainly due to the improved nutritional and water status of crops and to enhanced antioxidant metabolism and/or increased production of secondary metabolites in the plant tissues. However, the effectiveness of AMF in protecting their host plants against *Verticillium* spp. may vary depending on the environmental factors. Some environmental factors, such as the concentration of carbon dioxide in the atmosphere, the availability of soil water and the air and soil temperatures, are predicted to change drastically by the end of the century. The present paper discusses to what extent the climate change may influence the role of AMF in protecting crops against Verticillium-induced wilt, taking into account the current knowledge about the direct and indirect effects that the changing environment can exert on AMF communities in soils and on the symbiosis between crops and AMF, as well as on the development, incidence and impact of diseases caused by soil-borne pathogens.

## 1. Mycorrhizal Fungi Can Protect Crops against Soil-Borne Pathogens

Mycorrhizal fungi are terrestrial fungi that coinhabit the rhizosphere with many saprotrophic and pathogenic fungi, interacting with one other [1]. Mycorrhizal fungi colonise plant roots, forming ecologically and agronomically important symbioses called mycorrhizae, which are among the most common symbioses in nature. Among mycorrhizae, arbuscular mycorrhizas (interactions between plants and fungi in the phylum Glomeromycota) represent the most ancient symbiosis, as they were already formed by the first land plants at least 400 million years ago during the Ordovician-Devonian period [2] and being the most widespread fungal symbionts of current land plants, including crops.

Among all the benefits that arbuscular mycorrhizal fungi (AMF) can provide to their host plants is worth noting the protection against different pathogens and, especially, against soil-borne pathogens. There are numerous reports on the alleviation of damage caused by soil-borne necrotrophic pathogens, such as *Fusarium oxysporum* in strawberries [3], cucumbers [4] and chickpeas [5]; *Phythium aphanidermatum* in pawpaws [6]; *Armillaria mellea* in grapevines [7]; *Phytophthora parasitica* in tomatoes [8] or *Aphanomyces euteiches* in peas [9]. Guillon et al. [10] also observed a differential and systemic alteration in the expression of defence genes during the pathogenic interaction or mycorrhized beans infected with *Rhizoctonia solani*. Several mechanisms, acting together or separately, have been proposed to explain the bioprotection conferred by AMF to crops against soil-borne pathogens: enhanced plant nutrition, damage compensation, alterations in the root anatomy and longevity, competition between AMF and soil-borne pathogens for the colonisation sites in roots and for host photosynthates, activation of plant defences and changes in the populations of rhizospheric microorganisms [11]. Moreover, the effectiveness of mycorrhizal symbiosis for inducing the so-called mycorrhiza-induced resistance (MIR) in aerial organs against necrotrophic pathogens attacking roots is due to the quick activation of the jasmonate (JA)-dependent signalling pathway in their host plants [12].

*Verticillium* spp. are soil-borne ascomycete fungi with a very broad spectrum of host plants around the world. Although initially these fungi show biotrophic behaviours and do not produce severe reductions in plant performance, at later stages, they become necrotrophic and alter the hormonal balance, induce the synthesis of hydrogen peroxide and nitric oxide and cause the activation of defence genes in the plant, leading to the death of host cells [13]. Since almost 40 years ago, several studies have been performed to evaluate the role of mycorrhizal symbiosis on the performance of *Verticillium*-infected annual and perennial crops. Despite some exceptions [14,15], in most cases (Table 1), the presence of AMF colonising plant roots has proportional benefits to different crops undergoing *Verticillium* attacks. Those benefits can be specified in reduced disease development and severity [16,17,18,19,20,21], as well as in improved plant growth [16,18] and enhanced yield [16,17], as a consequence of better water status [17,20], higher photosynthetic rates [17,20] and enhanced defence reactions against the pathogen [17,22]. Most parts of these studies have been carried out under greenhouse conditions, applying AMF previously to the attack of the pathogen. Some of the debatable points of these studies may be (a) the use of a restricted soil volume and (b) the pre-inoculation of AMF, which may not exactly reflect the reality in the field. Therefore, those aspects may induce thinking that the results obtained under controlled conditions cannot be extrapolated to nature. In relation to the first observation (limited soil volume), it is important to note that any restriction for the free growth of the extraradical fungal hyphae will exert a negative influence on the primary benefit of AMF for the host plant: the increased exploitation of soil volume for water and mineral acquisition. Moreover, the pot size can also modify the tolerance of mycorrhized host plants to stresses [23]. Consequently, we can affirm that the benefits of AMF on *Verticillium*-infected plants cultivated in pots (Table 1) occurred despite that the growth conditions were not the most adequate for the optimal performance of mycorrhizal symbiosis. In relation to the pre-inoculation of AMF in many of the studies (Table 1), it has been demonstrated that the time of crop inoculation with AMF (before, simultaneously or after the attack of the pathogen) can strongly affect the efficacy of mycorrhizal symbiosis in the protection of the host plants [24]. The pre-inoculation with AMF can nullify or decrease the negative effects of soil pathogens on roots [25]. Since AMF and *Verticillium* spp. cohabitate in the soil, the pre-inoculation of seedlings with AMF in nurseries could be an advisable practice, at least for annual crops. Finally, it is worth noting that the conclusions derived from different assays with cotton cultivated in pots or field conditions with AMF pre-inoculated or simultaneously inoculated with *Verticillium dahliae* have always demonstrated the protective role of AMF for the plant (Table 1). In fact, when cotton was cultivated in pots under greenhouse conditions, the mycorrhizal association beneficed plant growth and decreased disease incidence; regardless, AMF were previously or simultaneously inoculated with *V. dahliae* [16,18]. Moreover, the simultaneous inoculation of AMF and *V. dahliae* in the field induced the expression of pathogenesis-related genes and lignin synthesis-related genes in the plant [22], thus supporting the protective effect of AMF observed in greenhouse-grown cotton.

## 2. Climate Change Can Influence the Development, Incidence and Impact of Crop Diseases

The traditional plant disease triangle represents a balanced figure in whose vertexes appear the three fundamental elements required for the development of the disease: (a) a susceptible plant, (b) a pathogen able to cause disease and (c) a favourable environment, which includes factors affecting the plant, the pathogen or even additional organisms, such as vectors. However, the environmental changes occurring nowadays are steadily shaping the relationship between plant growth and the associated diseases. In the case of diseases caused by pathogenic fungi, it has been demonstrated that the triangle shape is becoming skewed because of the pressure of the environmental component on plant growth, physiology and yield [26], which alters the period of higher susceptibility to pathogens [27], heightens the risk of disease prevalence [28] and may drive the resilience of crops [29]. Global warming is inducing higher levels of aggressiveness and resistance in some strains of plant pathogens and increases the risk of transference of those pathogens from agricultural ecosystems to natural flora [28]. Increased temperatures may also allow pathogens to overwinter in higher latitudes and favour the emergence of new pathogenic strains. In addition, changes in both the air and soil temperature and humidity affect both the virulence mechanisms displayed by the pathogens (the production of toxins and virulence proteins) and their reproduction and survival [30]. The climate change can also affect the diversity and geographical distribution of pathogenic fungi, the development of post-harvest diseases and the severity of the mycotoxins released [29] and may induce the evolution of cryptic and latent pathogens [31]. For all these reasons, plant pathogens, together with climate change and environmental degradation, are the three main threats to food security [28].

Predictions on the possible incidence of climate change on soil-borne pathogens and the incidence of the diseases they cause in crops are not clear. Gioria et al. [32] argued that the climate change will not influence significantly on the occurrence of late blight caused by *Phytophthora infestans*, white mould produced by *Sclerotinia sclerotium* and wilt caused by *Verticillium albo-atrum* in tomatoes. In agreement with this point of view, Zhou et al. [33] found that the severity of the disease caused by *Fusarium oxysporum* f. sp. *raphanin* and *Rhizoctonia solani* in *Arabidopsis* did not change under different concentrations of CO_2_ in the atmosphere. However, as these last authors commented, in a global context of climate change, disease incidence will also be affected by other parameters, such as elevated ozone, humidity, drought and increased temperature. Even some agronomic practices, such as fertilisation, can modulate the effects of environmental parameters. In a study performed with potatoes infected with *Phytophthora infestans*, Osswald et al. [34] observed that the C/N ratio was crucial for the resistance or susceptibility of potatoes to the pathogen. While a rise in CO_2_ (700 ppm) enhanced the resistance of a susceptible potato cultivar towards *P. infestans* due to an increased C/N-ratio in the leaves, N fertilisation nullified the enhanced resistance as a consequence of a reduced C/N ratio. It is expected that higher CO_2_ concentrations in the air will induce the accumulation of carbohydrates in plant tissues and the reduction in the levels of proteins and amino acids in the tissues of many plants, thus increasing the C/N ratio. This enhanced C/N ratio may improve the tolerance of crops against soil-borne pathogens but can also diminish the nutritional quality of crops. Increased CO_2_ in the air may also modulate the crop resistance against *V. dahliae* through changes in the balance between the jasmonate (JA) and salicylic acid (SA) signalling pathways. It has been observed that an elevation of the atmospheric CO_2_ concentration can suppress the JA pathway in favour of the SA pathway [35]. Gao et al. [36] observed that plants attacked by *V. dahliae* quickly activate the JA pathway in order to strengthen the defences detriment to growth. However, *V. dahliae* can counteract the priority for the JA signalling in order to favour the development and reproduction of plants instead of their defence [37]. More recently, Scholz et al. [13] postulated that, during the later necrotrophic phase, *V. dahliae* stimulates the host JA functions in order to promote host cell death, which suggests that the effect of *V. dahliae* on the host JA pathway may depend on the stages of the disease cycle. In summary, the effect that increased CO_2_ could exert on the tolerance of plants against *V. dahliae* appears uncertain; while an increased C/N-ratio could enhance plant resistance, the suppression of the JA pathway could reduce the ability of plants to fight against *V. dahliae* in the early stages of the infection.

Together with increased atmospheric CO_2_, other environmental factors linked to the climate change are erratic and extreme rainfalls and increased air and soil temperatures. The vulnerability of crops to the attacks of soil-borne pathogens can increase after the extreme drought and rainfall events, in part due to the reduction of the natural capacity of soil to suppress pathogens, a fact verified when plants infected with fungal species belonging to *Fusarium* and *Verticillium* genera have faced drought conditions [38]. Similarly, Wakelin et al. [39] were not optimistic about the consequences that drought associated to climate change will exert on the incidences of many of the diseases affecting key agricultural crops in New Zealand. Moreover, Calderón et al. [40] found that soil temperature was a key element to determine the disease development in olive cultivars, showing a distinct degree of susceptibility to defoliating and nondefoliating pathotypes of *V. dahliae*. Although the findings arisen from the assays performed under controlled conditions do not clarify the consequences of climate change on the diseases caused by soil-borne pathogens, a real fact is that the incidence of Verticillium wilt affecting sunflowers in the field has risen since 2013–2014 in several European countries, including Bulgaria, France, Italy, Spain and Ukraine [31]. This observation under natural conditions induces keeping aware and looking for tools in order to counteract the increasing incidence of Verticillium wilt affecting crops. As pointed out by Cheng et al. [41], most investigations into plant-pathogen interactions have been carried out in greenhouses or growth chambers with simple and static environmental setups, while, in nature, a static environment is the exception, and a modification in a given environmental factor is usually linked to changes in other environmental parameters. The same observation can be applied to the symbiotic relationships between crops and AMF under changing natural environmental conditions. However, this does not detract from the importance and credibility of the results obtained in the studies carried out under controlled greenhouse conditions, since the fact of setting certain environmental parameters to study the effects exerted by others make it easier to ascribe the cause of a specific effect.

The information, currently available and obtained from both field studies and assays under controlled greenhouse conditions, indicates that the rainfall regime and temperature may be more determining factors than the high CO_2_ in the air for the development, incidence and impact of crop diseases.

## 3. Climate Change Can Affect Mycorrhizal Fungal Communities and Mycorrhizal Fungal-Crop Interactions

Changing environmental conditions can affect mycorrhizal fungi both directly and indirectly through modifications induced in the physiology and metabolism of their host plants. While the effects of tropospheric O_3_ and atmospheric CO_2_ on mycorrhizal fungi seem to be completely indirect, the influence of warming, extreme rainfall events and nitrogen deposition can be both direct and indirect [42]. Vice versa, the direct effects of elevated temperature and nitrogen addition on AMF can affect, in the last instance, the composition and productivity of plant community [43].

To date, there is not conclusive information on the effect that elevated tropospheric ozone may exert of AMF in the soils. More long-term studies are needed to conclude the accumulative effects of ozone on the composition and activity of AMF communities in the soils. By contrast, many studies (see [35,42] for more details) have reported that elevated CO_2_ in the atmosphere often reduces the abundance of AMF belonging to the Gigasporaceae and promotes the presence of Glomeraceae, thus changing the structure of the mycorrhizal communities present in soils. The reduction or exclusion of Gigasporaceae from agricultural soils will also represent a loss of functional diversity for crops, being the nutrient uptake and the consequent increase in plant vigour some of the aspects most negatively affected, which could be in detriment of the compensation of damage caused by pathogens [44]. Nevertheless, this effect may be masked by the natural changes through time [45] and, also, by the dependency of different fungi on water availability and precipitation [46]. Elevated CO_2_ can also indirectly promote the mycorrhizal colonisation of plant roots as a consequence of the enhanced carbon allocation to roots [47,48], but the positive effect of elevated CO_2_ on root colonisation and the growth of extra-radical fungal hyphae has been mainly found in studies carried out in greenhouses rather than in the field [49]. Another indirect effect of climate change on AMF is due to changes in the soil properties and in the communities of other neighbouring microorganisms. As Cotton [42] documents in her review, the levels of some essential elements such as nitrogen, phosphorus, potassium and carbon in the soil and, also, the pH values can be modified by alterations in the temperature, rainfall and atmospheric composition. The exudates from the roots to the soil can also vary as a consequence of the influence exerted by environmental conditions on the plants. The interaction between AMF and other microorganisms may also play a key role for the functional diversity in agricultural soils [44].

The conclusions arisen from studies focused on the responses of AMF to increased temperature in the soil are also uncertain. Wilson et al. [50] concluded that the direct effect of increasing 3 °C of the temperature decreases AMF colonisation, and this appeared to be regionally consistent across the Mediterranean climate gradient. By contrast, Hawkes et al. [51] observed that soil warming induced the growth of the extra-radical hyphae, which, in the last instance, may favour the uptake of water and minerals by colonised roots. However, this enhanced growth of the extra-radical mycelium can vary among fungal species [52] and not always is associated with an improved mycorrhizal activity [53]. These last authors also summarised the results obtained in several studies whose objective was to elucidate the effect of rising temperatures on mycorrhizal communities; the most part of the investigations reported an enhanced abundance of AMF when the temperature increased. Nevertheless, the intraspecific variation in the limits of temperature for spore germination may modify the diversity of mycorrhizal species associated to roots, since the first fungus to colonise a plant usually becomes dominant in the later overall community [42].

Changes in water availability due to extreme rainfall or drought events can modify the diversity of AMF communities in soils, but once more, there is not a consensus among different studies to conclude if the diversity increases, decreases or remains unchanged [42]. One aspect that has not been widely investigated is the increased compaction linked to the loss of water in some types of soils and its impact on diversity, activity and symbiosis of AMF with plant roots. Compaction is a physical degradation of soil that implies the disturbance of its structure and the reduction of the size of the pores [54], and it is more frequent and severe in clay soils and can be aggravated by the use of machinery in agricultural soils. High soil compaction may limit the physical growth of fungal hyphae and deteriorate the integrity of the extraradical mycelium [55]. An intact extraradical mycelium can remain infective over long periods, even under suboptimal environmental conditions, and it represents the preferential type of AMF propagule that ensures an earlier and faster establishment of the mycorrhizal association in crops [44]. Moreover, both the reduced size of the pores in a compacted soils and the small air phase in waterlogged soils strongly limit the interactions between microorganisms via volatile organic compounds [38].

In summary, changes in the composition, diversity and abundance of the AMF communities as a consequence of adverse climatic events and/or inappropriate agricultural practices may alter feedback to the plant community, reducing the beneficial effects of AMF on plant fitness, productivity and protection against pathogens [44].

## 4. Mycorrhizal Protection of Crops Against Verticillium Wilt Under Climate Change Scenarios: A Perspective Based on the Limited Current Scientific Information

The current scientific information does not allow elucidating with precision the role that AMF will play in protecting crops against soil-borne pathogens under climate change scenarios. There are still many gaps that should be investigated. There is very scarce information on the direct effects that the environmental parameters linked to the changing climate exert on the soil-borne pathogens in general and on *Verticillium* spp. in particular. Moreover, as commented by Meisner and de Boer [38], basic knowledge about the direct effects that extreme weather events can exert on (a) the interactions among soil microorganisms, (b) the survival of microorganisms that induce pathogen suppression and (c) the recovery of the pathogen suppression are lacking in the literature. Most scientific documents report information on the behaviour of the dual interactions plant-pathogen or plant-AMF under changing environmental conditions. Therefore, it is necessary to look deep into the tripartite interaction crop- pathogen-AMF under scenarios of climate change in order to provide scientific evidence on the degree of bioprotection of crops by AMF against Verticillium wilt. Furthermore, taking an expression by Wipf et al. [56], “AMF are not alone” in the soil. They interact with other mutualistic root microorganisms, such as plant growth promoting rhizobacteria (PGPR), mostly with synergistic results [57]. In addition, the soil fauna is known to play key roles in shaping the microbial communities associated with plants, but its effect on the interaction between plants and rhizospheric microorganisms remains unclear under stressful conditions [58].

**Scheme 1:**
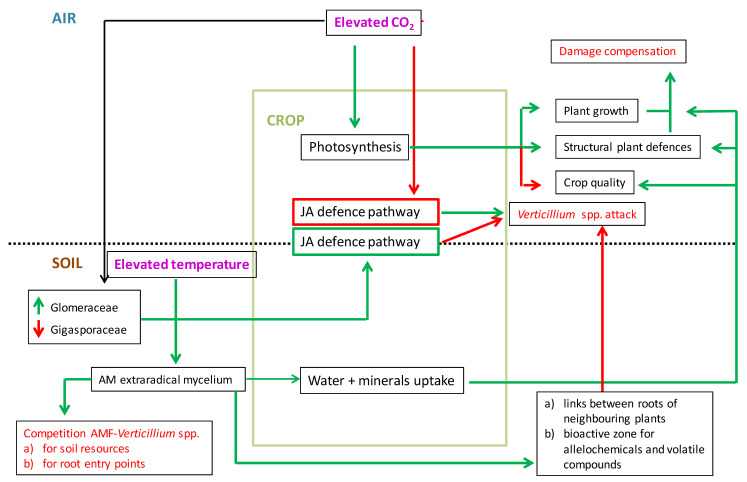
Hypothetical effects of elevated CO_2_ in the air and elevated soil temperature on the role of arbuscular mycorrhizal fungi (AMF) as bioprotectors of crops against Verticillium wilt. Green arrows indicate “induced or increased” and the red arrows indicate “avoided or decreased”. AM = arbuscular mycorrhizae; AMF = arbuscular mycorrhizal fungi; JA = Jasmonic acid.

**Scheme 2:**
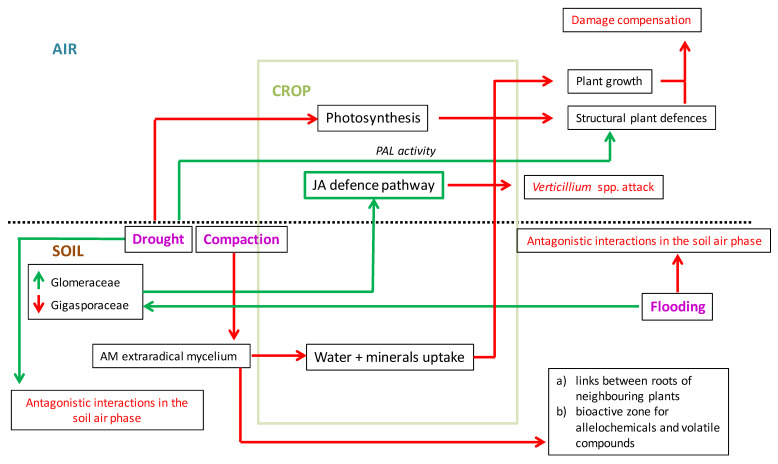
Hypothetical effect of elevated CO_2_ in the air and elevated soil temperature on the role of arbuscular mycorrhizal fungi (AMF) as bioprotectors of crops against Verticillium wilt modified by the irruption of erratic rainfall and severe drought periods. Green arrows indicate “induced or increased”, and red arrows indicate “avoided or decreased’. AM = arbuscular mycorrhizae; JA = Jasmonic acid; PAL = phenylalanine ammonia lyase.

Atmospheric CO_2_ concentrations have increased from about 280 ppm in the preindustrial era until the current 414.5 ppm [59]. Estimated models predict that the CO_2_ concentration will not stabilise but will continue increasing over the coming decades [60]. The models that predict CO_2_ concentrations of 1000 ppm by 2100 also estimate that the air temperature is likely to exceed 4 °C above preindustrial levels [61]. These constantly increasing levels of CO_2_ and temperatures in the air will be combined with periods of severe drought or extreme rainfalls [62]. Therefore, the hypothetical model then exposed is firstly based on the current information on the direct and indirect effects of elevated CO_2_ and temperature on crops and AMF and their possible consequences for the triple interaction crop, AMF, *Verticillium* spp. (Scheme 1). Scheme 2 represents how erratic rainfall and severe drought periods could modulate the presumed consequences of increasing air CO_2_ and temperatures in the interaction crop-AMF-*Verticillium* spp.

Under elevated CO_2_ in the atmosphere (Scheme 1), the photosynthetic rates of crops and, especially, those of C3 crops, will increase, thus producing more carbohydrates. This higher amount of sugars can improve crop growth and provide precursors for reinforcing structural barriers, for example. More vigorous crops with enhanced structural defences may be more resistant to pathogens attacks and compensate, at least in part, the damage caused by them, including *Verticillium* spp. However, crop quality may decrease because the elevated CO_2_ could both reduce the ratio between nutritional and caloric values of crops and intensify the already acute problem of micronutrient malnutrition even in the developing world [63]. It has been also demonstrated that elevated concentrations of CO_2_ in the air enhance SA-associated defences in detriment of the JA-associated defences [35] implied in the first reaction of plants to suppress *V. dahliae* infection [36]. Taking together, it could be suggested that elevated CO_2_ may exert a negative effect on crop defence against *V. dahliae* at the early stages of the infection but enhance the vigour of the plants to compensate for the negative effects of the pathogen on growth and yield.

Despite the disparity in the results obtained when studying the direct effect of climate change parameters on AMF in soils, there is a higher degree of consensus when affirming that (a) elevated CO_2_ will favour the presence of species belonging to the Glomeraceae family in detriment of those from Gigasporaceae family and (b) warming can induce the growth of the extraradical hyphae in the soil (Scheme 1). If so, it could be expected that AMF belonging to the Glomeraceae family could counteract the suppression of the JA defence pathway induced by the elevated atmospheric CO_2_ in crops, thus reinforcing the ability of crops to fight against *V. dahliae*. In addition, a higher presence of extraradical mycorrhizal hyphae could increase the tolerance of crops against *V. dahliae* through different ways: (a) by increasing the direct competition between AMF and *V. dahliae* for soil resources and root entry points, (b) by enhancing the uptake of water and soil minerals by roots, thus benefiting crop growth (and indirectly favouring a more balanced ratio of carbon/minerals and improving the quality of crops) and (c) by allowing in the field belowground interactions between individuals thanks to the mycelium shared by the roots of neighbouring plants. These common mycorrhizal networks (CMN) represent a promising tool for the biocontrol of crop pests, because they increase the bioactive zone for allelochemicals and volatile compounds in the soil (see [56] for more information).

The general picture explained in Scheme 1 can be strongly affected by the erratic and extreme rainfall events related to the climate change (Scheme 2). Severe drought will cause stomatal closure in crops in order to avoid high water loss by transpiration, and, consequently, the uptake of atmospheric CO_2_ will decrease, thus reducing the biosynthesis of sugars. In C3 plants, the efficiency of the photosynthesis will be also limited by an increased photorespiration process in warm and dry areas. Therefore, under prolonged drought, the growth and vigour of the crops will decrease, thus limiting the capacity of plants for compensating the damage caused by soil-borne pathogens in general and *Verticillium* spp. in particular. In this context, the improved uptake of soil water and minerals by AMF can be crucial for their hosts. In fact, it is usual that the beneficial effects of AMF for plants appear more evident when plants are undergoing a water deficit than when they are growing under an optimal water regime [55].

Another aspect to highlight is the big air phase in the dried soils, which facilitates the interactions between microorganisms via volatile organic compounds [38]. It has been found that AMF can alter the profiles of volatile organic compounds released by roots [64], and, in a recent investigation, Wonglom et al. [65] demonstrated that an endophytic fungus emitted volatile organic compounds involved in antifungal activity in the induction of defence responses and in the improvement of growth in lettuce. Therefore, it does not seem nonsensical to hypothesise that, when crops are undergoing drought periods, AMF may increase their resistance against *Verticillium* spp. through the production of volatile organic compounds that would easily diffuse into the soil. However, if the loss of water is accompanied by an increase in the compaction of the soil, fungal structures (mainly the extraradical mycelium) will be damaged. The disruption of the belowground fungal network can severely impair both the uptake of water and minerals and the exchange of signals between plants for increasing their resilience against pathogens. Moreover, the reduced size of the soil pores under compaction will impair the diffusion of volatile compounds.

As previously commented, extreme drought can reduce the natural capacity of soil to suppress pathogens, thus increasing the vulnerability of crops to the attacks of soil-borne pathogens [38]. Nevertheless, water deficit may also activate some mechanisms that enhance the tolerance of plants against *Verticillium* spp. Lignin deposition in root xylem can be induced under drought [66], a mechanism implied in the resistance of cotton plants against *V. dahliae* [67]. Similarly, mycorrhizal symbiosis induced an early lignin deposition in the stem xylem of pepper plants and increased the activity of the enzyme phenylalanine ammonia-lyase in roots, mechanisms possibly implied in the slowing down of the disease symptoms onset in plants are infected by *V. dahliae* [17]. This indicates that the association of crops with AMF may anticipate lignin deposition in the xylem of the crops, even before any drought period occurs. It would be very interesting to investigate if mycorrhization and water deficit can have a synergic effect in reinforcing the xylem through lignin deposition when both factors occur simultaneously or sequentially.

The erratic rainfall related to the climate change can also trigger flooding, which will strongly influence the antagonistic interactions between pathogens and other soil microorganism in the soil [38]. As explained by these last authors, under waterlogged conditions, interactions between microorganisms will mainly occur in the water phase of soils, thus limiting the interactions mediated by volatile organic compounds. Flooding can decrease the AMF colonisation of roots [68] and affect the diversity of AMF communities in the rhizosphere [69] in spite of which mycorrhizal symbiosis can improve flooding tolerance of host plants [70]. According to the findings of Wang et al. [69], mycorrhizal species belonging to Glomeraceae (those inducing the JA defence pathway in plants implied in stopping the early infection of *V. dahliae*) will predominate in flooded soils.

## 5. Conclusions and Perspective

The current scientific evidence offers an incomplete and fragmentary view on the role that AMF may play as bioprotectors agents of crops against Verticillium wilt under scenarios of climate change. Most of the conclusions were obtained from studies performed in a greenhouse and only managing one parameter related to climate change (mainly elevated CO_2_, drought or high temperature) applied to a dual interaction plant-pathogen or plant-AMF. The interactions between several climatic change parameters acting together or sequentially can strongly influence the final result. Moreover, in the field, there are many rhizospheric interactions among microorganisms (beneficial, pathogenic or neutral for plants) and fauna, which can modulate the behaviour of the triple-interaction ‘crop-*Verticillium* spp.-AMF’.

Despite the limited scientific evidence, it can be hypothesised that mycorrhizal fungi may be key players in the tolerance of crops against the consequences of *Verticillium* spp. infection in a future climate change scenario. Apart from the benefits that mycorrhizal symbiosis can exert for the hosts by favouring the uptake of water and minerals from the soil, thus improving plant growth, vigour and ability for compensating damage caused by *Verticillium* spp.; in the field, the extraradical mycelium is emerging as the key fungal structure for the resilience of the entire plant community. Increased, the growth of extraradical hyphae observed under elevated soil temperatures not only increases the competition with *Verticillium* spp. for soil resources and/or root contact points but, also, connects individuals of the community for transferring nutrients and signalling compounds among them, thus improving the tolerance of the whole community against abiotic and biotic stresses. Moreover, if elevated CO_2_ causes that, mycorrhizal species belonging to Glomeraceae are predominant in the soil and those AMF could counteract the diminished JA-pathway in plants, thus helping crops defend themselves against *V. dahliae* attacks.

Possibly the greatest danger to the efficacy of fungi as crop bioprotectors against Verticillium wilt in climate change scenarios lies in the deterioration of the extraradical mycelium. Apart from the disruption of the extraradical hyphae as a consequence of high compaction in dry clay soils, some agronomic practices can strongly aggravate this effect produced by severe drought periods. Among those agronomic practices that negatively influence on the belowground hyphal network are the use of heavy machinery that compacts the soil, the application of agricultural practices that greatly crumble the upper layers of the soil and the cultivation of crops that do not associate with AMF (those from Brassicaceae, for example) for long periods.

Genetic resistance is considered as the most economical approach to crop protection, so that the search of naturally occurring resistances has been a recurrent subject of investigation. The success of many genetic engineering programs focused on increasing the resistance of crops to diseases is highly subject to public acceptance. In a way, the association of the crop species with AMF represents a natural way of altering the gene expression, which can enhance crop tolerance against pathogens and environmental constraints.

## Figures and Tables

**Table 1:** Compendium of studies dealing with the effect of arbuscular mycorrhizal fungi (AMF) as bioprotectors of crops against *Verticillium dahliae.*

Crop	Growth Conditions	Application of AMF	Effects on Plants	Reference
Cotton	Greenhouse (pots)	Pre-inoculation	● Increased plant dry weight ● Reduced disease index ● Decreased levels of phenolics in root exudates	[18]
	Pots	Simultaneous	● Increased plant growth ● Advanced flowering ● Increased number of flowers and bolls ● Decreased disease incidence	[16]
	Field	Simultaneous	● Induced expression of pathogenesis-related genes and lignin synthesis-related genes	[22]
Pepper	Greenhouse (pots)	Pre-inoculation	● Delayed the appearance and development of the disease symptoms ● Increased chitinase and superoxide dismutase activity in roots ● Induced the lignification of xylem in stems ● Increased the antioxidant metabolism in leaves ● Improved plant water status ● Favoured the maintenance of photosynthesis ● Diminished the reduction of yield caused by *V. dahliae*	[17]
Strawberry	Greenhouse	Pre-inoculation	● Reduced disease development ● Increased stomatal conductance and transpiration rate ● Improved plant water relations ● Counteracted the decreased photochemical activity in susceptible cultivars	[20]
Olive	Greenhouse (pots)	Pre-inoculation	● Reduced dwarfing and leaf alterations ● Decreased presence of *Verticillium* in roots and stems	[21]
Tomato Pepper Eggplant	Growth chamber	Pre-inoculation	● Reduced wilt disease severity and microsclerotia number (AMF combined with humic acids and/or whey)	[19]
